# The role of autoantibodies in the neuropsychiatric manifestations of 22q11 deletion syndrome

**DOI:** 10.1017/neu.2025.10046

**Published:** 2025-11-26

**Authors:** Suzain Ali, Brad D. Pearce

**Affiliations:** Department Epidemiology, Rollins School of Public Health, Emory University, Atlanta, GA, USA

**Keywords:** 22q11DS, neuropsychiatric, autoantibodies, autoimmunity, schizophrenia

## Abstract

The 22q11.2 deletion syndrome (22q11DS) is a genetic disorder characterised by defined microdeletions at chromosome 22q11.2. These genetic changes lead to a variety of neurodevelopmental problems, including cognitive delays and a very high rate of symptoms on the autism and schizophrenia spectrum. The underlying mechanisms contributing to these neurodevelopmental manifestations remain poorly understood. In concert with these neurodevelopmental difficulties there are also immune system alterations, including autoimmunity. We hypothesise that immune dysfunction and the presence of circulating autoantibodies may play a role in the pathophysiology of these neuropsychiatric symptoms. In this review, we synthesise the diverse literature on autoantibodies in 22q11DS and propose mechanisms for a causative role of these autoantibodies in neurobehavioural problems such as psychosis and cognitive delays. This review highlights the importance of further research to explore the interaction between autoreactive antibodies and functional alterations in neurocircuitry function. Understanding this relationship may provide insight into the origins of psychiatric symptoms.

## Graphical abstract

**Figure:**
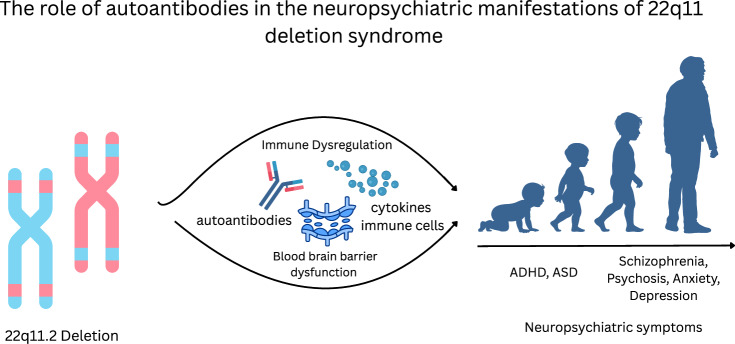

Summation

- Indirect evidence supports the concept that autoantibodies may be involved in the neuropsychiatric symptoms in 22q11DS
- Potential mechanisms include the direct action of anti-neural autoantibodies in the presence of increased blood–brain barrier permeability, elevated levels of soluble inflammatory mediators such as IL-6 and immune-mediated disturbance of thyroid hormones.
- Routine screening for autoantibodies is not yet standard practice for 22q11DS patients with neuropsychiatric symptoms

Limitations

- Many of the studies supporting this perspective did not directly study patients with 22q11DS
- The literature supportive of our perspective is a diverse mix of epidemiological association studies, clinical correlations, animal models, and *in vitro* experiments
- We cannot yet narrow down the target antigens responsible, and large-scale autoantibody screening is expensive

## Introduction

The 22q11 deletion syndrome (22q11DS) is a copy number variant disorder in which there is a hemizygous (1 copy) microdeletion in chromosome 22q11.2. In approximately 87% of patients, the deletion occurs in a well-defined 3 megabase (Mb) region containing 46 unambiguous genes (Maynard *et al*., 2002; Antshel *et al*., 2005). In approximately 10% of patients, there is nested deletion of approximately 1.5 Mb (Carlson *et al*., 1997; Shaikh *et al*., 2000). In addition to neurodevelopmental and neuropsychiatric manifestations, there are variable alterations in peripheral systems including cardiac lesions, parathyroid hormone deficiency, and immune abnormalities (Kobrynski and Sullivan, 2007; Karbarz, 2020). The purpose of this review is to present a cohesive argument that autoantibodies may contribute to some of the neuropsychiatric manifestations of 22q11DS. This perspective has received little attention and opens new territory for future investigations.

## Immune system in 22q11DS

Many of the immunological characteristics of 22q11DS have been determined and provide the background to support our novel premise that autoantibodies contribute to the neuropsychiatric manifestations of this disorder (Gennery *et al*., 2002; Chinen *et al*., 2003; Morsheimer *et al*., 2017; Costagliola *et al*., 2023). One of the challenges in linking autoantibodies to the neuropsychiatric aspects of 22q11DS is the significant variability among individuals, including the degree and characteristics of immune dysfunction. The intense clinical and research interest in the immunological aspects of 22q11DS will undoubtedly provide new data to refine the biological pathways that provide the nexus between immune and neurobehavioural alterations.

22q11DS is classified as a primary immunodeficiency disorder, but as described below, autoimmunity is also common. The immune deficiency is attributed to hypoplasia or aplasia of the thymus in 22q11DS due to incomplete embryonic development of the third and fourth pharyngeal arch structures (Gennery, 2007). Nevertheless, severe immunodeficiency and complete lack of the thymus turns out to be quite rare (< 0.5%) (Ryan *et al*., 1997), but on average T-cell counts are lower than in the general population despite considerable overlap (Chinen *et al*., 2003). 22q11DS is not simply a T-cell deficiency disease, and multiple branches of the immune system are abnormal. This is not surprising considering the number of genes that are affected, and the intertwined nature of immune responses (Morsheimer *et al*., 2017).

Patients with 22q11DS are predisposed to autoimmunity (Gennery *et al*., 2002; Davies *et al*., 2003). The mechanisms by which immune tolerance to self-antigens are disrupted in 22q11DS are beginning to be defined (Davies, 2013). Normally, immune tolerance is maintained through both central and peripheral mechanisms. Central self-tolerance occurs in the thymus by deleting self-reactive T cells (negative selection). Peripheral tolerance is exerted by multiple processes to assure that self-reactive T cells become functionally unresponsive or are deleted. Additionally, peripheral dendritic cells also play an important role in maintaining peripheral tolerance (Legitimo *et al*., 2020). In 22q11DS thymic insufficiency results in impaired generation of CD4+CD25+ regulatory T cells (Treg cells) which are essential for maintaining tolerance, which is one factor for the enhanced likelihood of developing an autoimmune disease (Davies, 2013). 22q11 deletion syndrome patients display reduced numbers of dendritic cells, which could contribute to increased vulnerability to infections and autoimmune disorders (Legitimo, 2020; Legitimo *et al*., 2020). A reduced T-cell receptor repertoire as well as homeostatic T-cell proliferation may also play a role in the predisposition to autoimmunity (Davies, 2013; Ferrando-Martinez *et al*., 2014). The syndrome is also associated with increased percentages of inflammatory Th1 and Th17 T cells (Vergaelen *et al*., 2018). Furthermore, the persistence of microbial antigens, coupled with an ineffective immune response, can give rise to the phenomenon of molecular mimicry that could contribute to autoimmune diseases (Costagliola *et al*., 2023).

While much of the focus has been on T-cell defects, abnormalities in B-cells and humoural immunity are also commonly reported (Smith *et al*., 1998; Gennery *et al*., 2002; Biggs *et al*., 2023). As described below, the production of autoreactive antibodies is an important component of autoimmune phenomena in 22q11DS, but its relationship to neuropsychiatric symptoms has received little attention.

## Neuropsychiatric manifestations of 22q11DS. Is there a role for autoantibodies?

We propose that increased levels of circulating autoantibodies cause disruption of brain circuitry in 22q11DS. Neurobehavioural difficulties are common in 22q11DS and include learning disabilities as well as an exceptionally high rate of autism spectrum disorders (ASD) and schizophrenia (Fiksinski *et al*., 2023). Approximately 14 – 50% of children with 22q11DS meet diagnostic criteria for ASD and about 30% of individuals develop schizophrenia (Karayiorgou and Gogos, 2004; Fine *et al*., 2005; Antshel *et al*., 2007; Green *et al*., 2009; Bassett *et al*., 2017). Investigations of the cellular and molecular mechanisms leading to the altered neurodevelopmental trajectory in 22q11DS have garnered broad interest because analogous mechanisms may play a role in the pathophysiology, including the more common forms that are not specifically related to an underlying genetic syndrome.

Individuals with 22q11 deletion syndrome have an increased risk of juvenile idiopathic arthritis, coeliac disease, autoimmune cytopenia, skin conditions, type 1 diabetes, and hyper or hypo-thyroidism (McDonald-McGinn *et al*., 2015; Biggs *et al*., 2023). Table 1 shows the wide variety of peripheral autoantigens that have been implicated in 22q11DS. For this table we systematically extracted the published literature (in English) for studies published since 1968 for autoimmune diseases in 22q11DS in which a distinct autoantibody was detected. Furthermore, autoantibodies have been detected in 22q11DS patients who have not been yet diagnosed with an autoimmune disease. Lima et al., (Lima *et al*., 2011) conducted a comprehensive autoantibody assay targeting various organ-specific antibodies such as the thyroid, adrenal glands, and the parathyroid gland along with general antibody markers such as anti-nuclear antibodies (ANA). Their findings revealed that 47% of study patients had a wide range of autoantibodies irrespective of whether they were diagnosed with an autoimmune disease.

**Table 1.** Autoantibodies in autoimmune diseases in 22q11.2 deletion syndrome retrospective or prospective cohort, case–control and cross-sectional studies that reported autoimmune diseases with positive autoantibodies among individuals with 22q11.2DS were selected

Disease	Autoantibody	Author, Year
Immune thrombocytopenia purpura (ITP)	Anti-platelet antibody	(Tison *et al*., 2011) (Patel *et al*., 2024) (Gennery *et al*., 2002)
Coeliac disease	Tissue transglutaminase (tTG) antibody	(Giardino *et al*., 2014) (Lima *et al*., 2011)
Grave’s disease	TSH receptor antibody Anti-thyroperoxidase antibody Anti-thyroglobulin antibody Thyrotropin binding inhibitory immunoglobulin	(Choi *et al*., 2005) (Ricci *et al*., 2022) (Stagi *et al*., 2010) (Crowley *et al*., 2022) (Lin *et al*., 2022) (Denkboy *et al*., 2023)
Hashimoto’s thyroiditis	Anti-thyroglobulin antibody Anti-microsome antibody	(Choi *et al*., 2005) (Ricci *et al*., 2022) (Lima *et al*., 2011) (Tison *et al*., 2011) (Sedivá *et al*., 2005) (Crowley *et al*., 2022)
Immune neutropenia/pancytopenia	Anti-neutrophil antibody/anti-granulocyte antibody	(Patel *et al*., 2024) (Tison *et al*., 2011)
Raynaud’s disease	Anti-smooth muscle antibody Rheumatoid factor antibody Cardiolipin antibody Anti-endothelial antibody Thyroid microsomal antibody Antinuclear antibody	(Lima *et al*., 2011) (Gennery *et al*., 2002)
Pernicious anaemia	Intrinsic factor antibody	(Lima *et al*., 2011) (Grinde *et al*., 2020)
Atrophic gastritis	Parietal cell antibody	(Lima *et al*., 2011) (Grinde *et al*., 2020)
Autoimmune haemolytic anaemia	Coombs positive (antiglobulin test)	(Patel *et al*., 2024) (Tison *et al*., 2011)
Evans syndrome	Rheumatoid factor antibody	(Gennery *et al*., 2002)

## Specific mechanism by which autoantibodies can cause psychiatric symptoms in 22q11DS

**Figure 1.**
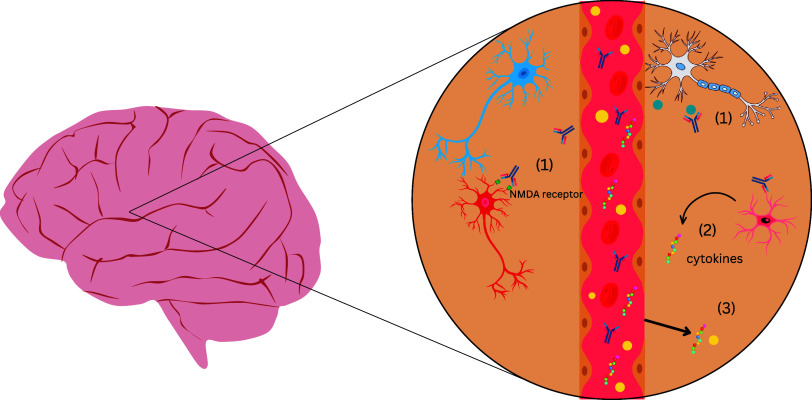
Schematic representation of proposed mechanisms by which autoantibodies may contribute to neuropsychiatric symptoms in 22q11.2 Deletion Syndrome. This figure depicts a blood vessel surrounded by brain parenchyma, highlighting potential immune-mediated mechanisms in 22q11.2 deletion syndrome. When the BBB is compromised, (1) autoantibodies that enter the CNS can bind neuronal receptors (e.g., NMDA receptors) and soluble antigens (e.g., LGI-1), disrupting synaptic signaling; (2) glial cells (microglia, astrocytes) are activated by autoantibodies and release pro-inflammatory cytokines such as IL-6 and TNF-α; and (3) the leaky BBB permits peripheral cytokines and immune cells to enter the CNS, propagating neuroinflammation. Together, these processes may contribute to aberrant synaptic pruning, neuronal dysfunction, and neuropsychiatric symptoms.

Autoantibodies in 22q11DS can potentially cross the blood–brain barrier (BBB) in the setting of increased permeability. As elaborated below, there are several stream of evidence indicating the structural and functional compromise of the BBB in 22q11DS. Once in the CNS, these autoantibodies may interact with neuronal or glial cells, leading to microglial activation, synaptic dysfunction, and cytokine release, potentially resulting in neuropsychiatric symptoms (Figure 1). Despite numerous studies testing for autoreactive antibodies against various peripheral antigens in 22q11DS (Table 1), there has been very little consideration of antibodies reacting with brain tissue.

We recognise that direct causal links between 22q11DS, autoimmunity and neuropsychiatric disorders is a novel and untested concept. Many of the proposed mechanistic links are based on associations and indirect evidence rather than controlled experimental studies. However, insights from established neuropsychiatric disorders and autoimmune disorders affecting the brain provide a framework for understanding these potential connections. Investigators have proposed various mechanisms by which autoreactive cells or antibodies are in causal chains leading to these neurobehavioural disorders. Li et al., provide an in-depth review of how the direct pathogenicity of anti- GABAaR antibodies cause GABAaR encephalitis (Li *et al*., 2024). This disease presents a complex neuropsychiatric profile that includes seizures, sleep disorders, and cognitive impairment. The role of autoantibodies has been established in this disease by a confluence of techniques including the production of human monoclonal antibodies from patients that stain relevant brain regions, the recapitulation of the human disease and animal models, and detailed in vitro studies including electrophysiological changes (Li *et al*., 2024). These studies provide a guide by which the role of autoantibodies in psychiatric symptoms of 22q11DS can be established.

Such studies are lacking for 22q11DS and hence support for this hypothesis derives from other disorders that have overlapping neuropsychiatric symptoms with 22q11DS. The immune abnormalities and susceptibility to autoimmunity in 22q11DS have parallels in ASD and schizophrenia (Atladottir *et al*., 2009; Patterson, 2009; Benros *et al*., 2014; Edmiston *et al*., 2017b; Villarreal *et al*., 2024)

Autoantibodies directed against neural antigens have been detected in ASD using a variety of techniques (Wills *et al*., 2007; Li *et al*., 2024). Most epidemiological and experimental animal studies emphasise maternal autoantibodies in the risk for ASD among the offspring (Edmiston *et al*., 2017a; Chen *et al*., 2023), which has limited direct relevance to 22q11DS. For probands with ASD, there is some evidence of an increased burden of autoantibodies compared to neurotypical individuals. A recent systematic review underscores the diversity of autoreactive immunoglobulins among individuals with ASD (Zou *et al*., 2020). This however does not prove causation, and presence of autoantibodies in ASD may well be intermingled with genetic and environmental factors.

The role of the immune system in schizophrenia pathogenesis is supported by a plethora of data, including studies of specific infections, high rates of certain autoimmune diseases, and various laboratory tests of immune molecules as well as animal models (Lesh *et al*., 2018; Yue *et al*., 2021). The epidemiological association of autoantibodies in schizophrenia is supported by a large literature including a meta-analysis (Ezeoke *et al*., 2013a; Ezeoke *et al*., 2013b). These studies in schizophrenia provide clues to a potential connection between autoantibodies in 22q11DS and psychotic symptoms.

Mechanistic studies are beginning to reveal the pathophysiological processes by which autoreactive immunoglobulins can lead to psychosis. One area of coalescence between various theories of psychosis and schizophrenia concerns dysfunction at the NMDA receptor (NMDAR) (Sherman *et al*., 1991; Akbarian *et al*., 1996; Duncan *et al*., 1999; Lahti *et al*., 1999; Newcomer *et al*., 1999; Tamminga, 1999; Meador-Woodruff and Healy, 2000). The possible role of autoreactive antibodies against the NMDAR in schizophrenia has been gaining credence, based in part on the now established role of these autoantibodies in causing anti-NMDAR encephalitis (Dalmau *et al*., 2008; Zandi *et al*., 2011; Pollak *et al*., 2014; Kayser and Dalmau, 2016; Jezequel *et al*., 2017). While this particular encephalitis is rare, it is strongly associated with psychosis. A recent case series found that 73% of persons with anti-NMDAR encephalitis had psychosis (Warren *et al*., 2021). A causal connection of anti-NMDAR antibodies with psychosis and other neurobehavioural symptoms in this disorder is further supported by improvement with immunotherapy (Pollak *et al*., 2020) and various animal models (Maudes *et al*., 2025). Since the mechanism for psychosis in anti-NMDAR encephalitis is binding of the autoantibody to the NMDAR, which disrupts glutamatergic neurotransmission, this disorder underscores the potential importance of anti-neural antibodies in at least some of the symptoms that also occur 22q11DS (Figure 1).

In the common form of schizophrenia and other psychotic disorder where neither encephalitis nor a definitive genetic disease has not been diagnosed, the significance of autoantibodies has been less straightforward. However, the inconsistencies in literature on the prevalence of anti-NMDAR antibodies are beginning to be resolved. It is increasingly clear that refinement of experimental approaches has been crucial to this understanding. For example, Cullin et al., (Cullen *et al*., 2021) performed a meta-analysis of the prevalence of anti-NMDAR antibodies in schizophrenia and found a strong association OR 4·43 [95% CI, 1·73 to 11·36] only when considering live cell assays. This implies that the search for anti-NMDAR antibodies in 22q11DS will require specific attention to the assay details and that a negative result does not necessarily imply that the autoantibodies are absent.

Laboratory studies will also be important in discerning the potential role of autoantibodies in 22q11DS. For anti-NMDAR in schizophrenia, Jezequel et al., screened 48 schizophrenia patients and 104 healthy controls for the presence of anti-NMDAR antibodies (Jezequel *et al*., 2017). The authors concluded that approximately 20% of the patients with schizophrenia had detectable anti-NMDAR antibodies in contrast to approximately 3% of healthy controls. Using a series of controlled in vitro experiments, they discovered that the antibodies generated from the schizophrenia patients differed in character from the antibodies generated by the healthy controls, despite similar titres on the best-validated live cell assay for anti-NMDAR binding. Specifically, the antibodies from schizophrenia patients caused destabilisation of synaptic NMDARs and their interacting partner, EphB2R. This neurophysiological alteration at the synapse was not seen among healthy patients who also had anti-NMDAR binding in the same assay. This underscores the importance of functional testing of anti-NMDAR antibodies in 22q11DS. One way this can be achieved is by using patient-specific neurons generated from iPSCs for both binding and functional studies.

Brain soluble proteins involved in intercellular communication have also been targeted by autoantibodies giving rise to neuropsychiatric manifestations that share some features with 22q11DS (Li *et al*., 2024). For example, in anti-LGI-1 limbic encephalitis 59% of patients presented with initial psychotic symptoms (Yi *et al*., 2025). In addition to positive symptoms such as hallucinations, delusions, and disorganised speech, patients also have cognitive difficulties and negative symptoms such as apathy. Seizures are also common in both anti-LG-1 encephalitis and in 22q11DS (Eaton *et al*., 2019; Baudin *et al*., 2021; Li *et al*., 2024). The LG-1 protein binds to a variety of receptors in the brain, and antibodies against this protein have been proposed to disrupt potassium channels and neurotransmission transmission through AMPA receptors (Baudin *et al*., 2021). Moreover, there is growing evidence that autoantibodies do not necessarily cause overt symptoms in many cases but rather may shape the symptom profile among vulnerable individuals (Li *et al*., 2024). A role for autoantibodies in the neuropsychiatric manifestations of 22q11DS certainly does not imply that the under-expression of genes within the 22q11 deleted region in neurons or glia is unimportant. While this conventional view remains valid, the presence of autoantibodies may help shape or exacerbate certain behavioural characteristics of the disorder.

As described above (Table 1), autoantibodies against a variety of tissues are fairly common in 22q11DS. Surprisingly, there is a lack of studies on 22q11DS that have expressly studied autoreactive antibodies against neural tissue and their relationship to psychiatric symptoms. This underscores the importance of screening for specific neurological autoantibodies in patients with 22q11 deletion syndrome who present with psychiatric symptoms. There is a need to combine immunological techniques with cutting-edge techniques to investigate antibody-mediated alterations in neuronal circuits, synapses, synaptic receptor function, and receptor localisation/trafficking. This could allow us to develop targeted therapies to these autoantibodies and establish an association between autoantibodies and neuropsychiatric symptoms in 22q11 deletion syndrome. A novel aspect of our perspective is that the combination of autoantibodies and BBB disruption in 22q11DS drive the neurobehavioural manifestations (Figure 1)

## Role of BBB permeability and soluble proteins

Disruption of the BBB in 22q11DS could play a dual role in autoantibody-mediated neurodevelopmental injury. It could allow antigens that normally are immune-privileged within the BBB to leak into the bloodstream and be recognised by peripheral immune cells. Even if this does not occur, autoantibodies associated with autoimmune diseases present in the peripheral circulation could more easily enter the CNS compartment via the leaky BBB.

Experiments by Crockett et al.(Crockett *et al*., 2021) used human BBB like endothelial cells derived from induced pluripotent stem cells (iPSCs) of schizophrenia-diagnosed 22q11 deletion syndrome (22q11DS) patients. These cells were compared to age- and sex-matched healthy controls. The findings revealed that BBB integrity was significantly compromised in schizophrenia-diagnosed 22q11DS patient cells. Considering that the claudin-5 gene is a crucial component of BBB integrity and is among the hemi-deleted genes in 22q11DS, the finding of Crocket al. that the localisation of this protein was highly disorganised in the 22q11DS cells also points to BBB disruption. This study also examined a mouse model of 22q11DS and reported evidence of extravascular leakage across the BBB.

Li *et al*. (2021) conducted similar experiments using iPSC-derived brain microvascular endothelial cells from schizophrenia-diagnosed 22q11DS patients and compared them to healthy controls to assess BBB integrity. Notably, 22q11DS patients exhibited reduced VEGF and CRKL signalling, both of which are critical for maintaining BBB stability (Li *et al*., 2021). Indeed, a recent study examining BBB deficits in cells derived from patients with 22q11DS found that restoring CRKL function pharmacologically improved BBB permeability (Li *et al*., 2023). This raises the possibility of drugs that could improve the BBB leakiness in 22q11DS and prevent the entry of autoantibodies into the brain parenchyma.

Taler *et al*. (2022) investigated age-related changes in BBB permeability in individuals with 22q11DS. Their findings revealed significantly elevated biomarkers indicating increased BBB permeability in older 22q11DS patients compared to younger individuals and healthy controls. While this could imply that age-related changes in the BBB correspond to the latent development of psychosis in this patient population, the study by Taler et al., argued against this mechanism in that they found that psychotic and non-psychotic 22q11DS patients had similar deficits in BBB permeability. However, since this study did not consider autoantibodies or differentiate those with and without autoimmune indices, autoantibodies may be an important second factor interacting with BBB disruption to cause psychosis. Therefore, our core hypothesis that the combination of autoantibodies and BBB permeability are important for neuropsychiatric illness remains valid.

Mechanistically, there is evidence that the overproduction of certain soluble mediators that target the BBB could be responsible for disrupted BBB permeability and possibly neuropsychiatric symptoms in 22q11DS (Mekori-Domachevsky *et al*., 2017; Crockett *et al*., 2021; Frusone *et al*., 2024). Individuals with 22q11DS demonstrate elevated levels of cytokines, including interleukin-12 (IL-12), IL-6, the IL-6/IL-10 ratio, IP-10 and TNF-*α*; and some of these cytokines corelate with autistic behaviours in these patients (Ross *et al*., 2013; Aresvik *et al*., 2016; Mekori-Domachevsky *et al*., 2017; Frusone *et al*., 2024). IL-6 is a key regulator of Th17 cell differentiation, a pro-inflammatory T-helper subset implicated in neuroinflammation and autoimmunity. Vergaelen et al., reported increased Th17 cell levels in 22q11DS individuals with neuropsychiatric symptoms, suggesting that IL-6-mediated Th17 activation may contribute to the development of psychosis and cognitive impairments in this population (Vergaelen *et al*., 2018). These alterations suggest a state of chronic immune activation that may contribute to BBB disruption and the emergence of psychotic symptoms The connection between specific cytokines and BBB disruption in 22q11DS warrants more direct experimental verification by in vitro studies and animal models.

As depicted in Figure 1, glial activation in the context of an autoimmune response is hypothesised as one mechanism connecting autoimmunity to psychiatric symptoms in 22q11DS. Pro-inflammatory mediators such as IL-6, TNF-alpha, IL-1beta, released by activated glial cells, have been shown to disrupt tight junction integrity and increase paracellular permeability of the BBB (Ronaldson and Davis, 2020; Takata *et al*., 2021). Additionally, aberrant microglial activation leads to excessive synaptic pruning and neuroinflammation, while astrocyte dysfunction impairs synaptic support (Crockett *et al*., 2021; Menghi *et al*., 2023). Considering that some cytokines themselves disrupt the BBB there could be a feedforward loop in 22q11DS in which inflammatory cytokines cause the BBB to be more permeable to immune molecules, which in turn induce glia-derived cytokines responsible for altering neurocircuitry function and leading to neuropsychiatric symptoms as well as increased BBB permeability. Possible mediators of this process implicated in autoantibody-mediated brain disorders include IL-1β, CCL2 and IL-17A (Platt *et al*., 2017). There are several gaps in the literature needed to establish a pathophysiological nexus between autoantibodies, inflammatory mediators, glial cells and BBB disruption in driving neuropsychiatric manifestations of 22q11DS. Indeed, other more indirect mechanisms need to be considered, such as the role of disrupted neuroactive hormones in the context of autoimmunity.

## Role of hormones

Hormonal changes in 22q11DS are possible intermediates or co-factors in autoimmune-mediated psychiatric sequelae. Autoimmune thyroid disease, particularly Hashimoto’s thyroiditis (HT), is highly prevalent in 22q11DS (Table 1). Hashimoto’s encephalopathy (HE) is a steroid-responsive neuropsychiatric disorder associated with autoimmune thyroid disease, characterised by cognitive impairment, seizures and altered consciousness (Canton *et al*., 2000; Ferracci and Carnevale, 2006). A temporal association was observed between HT and the onset of neuropsychiatric or encephalitic symptoms, which typically appeared at or after the diagnosis of HT. This relationship may be particularly relevant in patients with 22q11 deletion syndrome (22q11DS), where HE could contribute to neuroinflammation and the development of psychotic symptoms (Canton *et al*., 2000; Ferracci and Carnevale, 2006). Patients with HE often exhibit elevated levels of anti-thyroid antibodies, including anti-thyroid peroxidase (anti-TPO) and anti-thyroglobulin antibodies, which can cross-react with brain structures, leading to cognitive impairment and a range of neuropsychiatric manifestations (Churilov *et al*., 2019). Interestingly, HE can develop regardless of thyroid function status (euthyroid, hypothyroid, or hyperthyroid), suggesting that it is the autoimmune process rather than hormone imbalance could be mediating the neuroinflammation (Churilov *et al*., 2019). Given the high prevalence of psychotic symptoms in individuals with 22q11DS, and the established association between thyroid dysfunction and 22q11DS, HE (perhaps in a less overt form) may serve as a potential underlying mechanism contributing to these psychotic manifestations.

In 22q11DS, parathyroid hypoplasia and subsequent diminished parathyroid hormone reserve leads to a variable predisposition to low or borderline-low serum calcium (Cuneo *et al*., 1997; Ryan *et al*., 1997; McDonald-McGinn *et al*., 1999; Taylor *et al*., 2003; Hieronimus *et al*., 2006). In 22q11DS, hypoparathyroidism correlated with autoimmune diseases (Lima *et al*., 2011). Moreover, calcium regulation plays a major role in neurodevelopment and synaptic plasticity, and hypocalcaemia in 22q11DS has been associated with adverse neurobehavioural parameters. (Greer and Greenberg, 2008; Lohmann, 2009; Sadakata and Furuichi, 2010; Muldoon *et al*., 2015). Thus, hypocalcaemia may exacerbate the adverse effects of autoantibodies on neurocircuitry.

## Conclusions and caveats

There is substantial indirect evidence that autoimmunity, and particularly autoantibodies, are well poised as pathophysiological mediators of the dramatically elevated psychiatric symptoms observed in 22q1DS. This connection is far from being proven. If autoantibodies are involved, it is likely that they act in conjunction with facets of the disease that are more directly connected to hemideletion of genes in the disorder.

Whatever the connection is between autoantibodies and psychiatric illness in 22q11DS, there is a need for more targeted association studies, including those focused on brain antigens and autoantibodies in the blood and cerebral spinal fluid. This includes consideration of confounding variables, and appropriate selection of controls, which could include healthy controls as well as those with autoantibody conditions that are not due to 22q11DS. Another possibility is to test for the higher prevalence of anti-neural autoantibodies among patients with 22q11DS who exhibit neuropsychiatric symptoms compared to those who do not.

Well-controlled laboratory experiments will be essential to establish a direct causal mechanism and elucidate the specific cellular and molecular pathways that are responsible. Considering the complex interplay between CNS vulnerability due to the direct genetic component (22q11.2 hemideletion), autoimmune phenomena and hormonal changes in 22q11DS, a multipronged approach will be needed to support our core hypotheses. Relevant studies should include animal models, in *vitro studies* (e.g. using IPSC-generated neural cells), and human investigations. Ultimately, the goal is patient-specific interventions that will improve both immune and psychiatric sequelae in this important patient group.
